# High-Pressure Density Measurements and Modeling of Hydrocarbons: 1-Hexene, 2,4-Dimethylhexane, 2,2,4-Trimethylpentane, and *n*-Heptane

**DOI:** 10.1007/s10765-026-03823-6

**Published:** 2026-09-06

**Authors:** Dennis Alt, Bennet Hopp, Jens Staubach, Hans Hasse, Simon Stephan

**Affiliations:** 1 Molecular Thermodynamics Group (MTG), RPTU Kaiserslautern, 67663 Kaiserslautern, Germany; 2 Laboratory of Heat and Mass Transfer (WSÜ), OVGU Magdeburg, 39106 Magdeburg, Germany; 3 Laboratory of Engineering Thermodynamics (LTD), RPTU Kaiserslautern, 67663 Kaiserslautern, Germany

**Keywords:** Extreme conditions, Measurements, Equation of state modeling, Density

## Abstract

Liquid densities of the hydrocarbons 1-hexene, 2,4-dimethylhexane, 2,2,4-trimethylpentane, and *n*-heptane were measured over a wide range of temperatures (293.15 K to 393.15 K) and pressures (up to 120 MPa), substantially extending the state range covered by previously available literature data. The density data were modeled using both an empirical correlation as well as a molecular-based equation of state (EOS). For each substance, an empirical *Tammann–Tait* correlation was developed that reproduces the new measurements with an average absolute deviation (AAD) of about 0.01%. Comparison with literature data shows good agreement for all four substances. Liquid densities calculated from the SAFT-VR Mie EOS were benchmarked against both the new data and literature data, yielding deviations typically below 0.4% which supports the predictive capability of this EOS across the studied conditions.

**Supplementary Information:**

The online version contains supplementary material available at https://doi.org/10.1007/s10765-026-03823-6.

## Introduction

Accurate liquid density data for hydrocarbons at elevated pressures are essential for a wide range of technical applications and industrial processes [1–3]. Several of the compounds studied here—1-hexene, 2,2,4-trimethylpentane, and *n*-heptane—find applications as fuel components and additives in both conventional and synthetic fuels [4–8]. Pressures within fuel-injection systems can reach up to 250 MPa [9]. 1-Hexene also serves as a co-monomer in the production of polyethylene, where high-pressure conditions also prevail during polymer extrusion [4]. *n*-Heptane and 2,2,4-trimethylpentane are further employed as primary reference fuels for determining gasoline knock ratings [10]. Linear and branched alkanes are also key constituents of lubricants, where tribological contacts can experience local pressures exceeding 1000 MPa [11–13]. Beyond their direct industrial relevance, reliable density measurements are crucial for the development and parameterization of fundamental equations of state, EOS [14–17], which can be used for describing a large range of thermodynamic properties based on a thermodynamic potential [18].

Several measurement techniques are available for determining liquid densities, including hydrometers, pycnometers, and vibrating-tube densimeters [19–22]. Among these, the vibrating-tube densimeter is a well-established method, particularly suited for measurements at elevated pressures. It offers rapid measurement capabilities and high accuracy over a broad range of temperatures and pressures [23–25]. In this method, the fluid density is determined by measuring the oscillation period of a U-shaped tube filled with the sample. As a relative measurement technique, it requires calibration with reference fluids of known density. Consequently, the accuracy of the obtained density values is directly linked to the accuracy of the calibration standards used [26]. In this work, the liquid densities of 1-hexene, 2,4-dimethylhexane, *n*-heptane, and 2,2,4-trimethylpentane were measured at pressures up to 120 MPa and temperatures between 293.15 K and 393.15 K using a vibrating-tube densimeter. *n*-hexane and *n*-octane were employed as calibration fluids. The new experimental data complement and substantially extend the existing dataset, particularly in the high-pressure regime. Where possible, the results were compared with literature data. New, component-specific empirical correlations were developed to accurately represent the experimental density data obtained in this study.

Equations of state (EOS) are an important tool for the calculation and prediction of thermodynamic properties. In particular, molecular-based EOS have gained increasing attention, as they establish a connection between macroscopic thermodynamic behavior and the underlying molecular structure of the fluid [27–34]. Molecular-based EOS often enable extrapolations beyond the range of states considered during parameterization—including metastable states [35, 36] and states at extreme pressure [37–40]. Among molecular-based EOS, equations of state based on the statistical associating fluid theory (SAFT) represent the current state-of-the-art and are widely used especially in academic contexts. In particular, the SAFT-VR Mie EOS [41] has been shown to yield excellent performance in describing thermodynamic properties at high pressures and temperatures [16, 28, 42–44]. In this work, the SAFT-VR Mie EOS was deployed for describing the density of the four considered substances.

## Experimental Methods

### Chemicals and Sample Preparation

All chemicals were used without further purification. *n*-Hexane and *n*-octane were used as calibration fluids (Table 1).

**Table 1:** Suppliers and purities of the chemicals used in this work

Substance	CAS reg. no	Supplier	Mass fraction purity^a^ (%)
*n*-Hexane	110-54-3	Merck Supelco	≥ 99.0
*n*-Octane	111-65-9	Sigma-Aldrich	99.3
1-Hexene	592-41-6	Thermo Scientific	99.5
2,4-Dimethylhexane	589-43-5	Fisher Scientific	99.9
2,2,4-Trimethylpentane	540-84-1	Thermo Scientific	99.9
*n*-Heptane	142-82-5	Fisher Scientific	99.8

^a^As specified by the supplier

### Measurements

#### Experimental Setup and Procedure

**Fig. 1:**
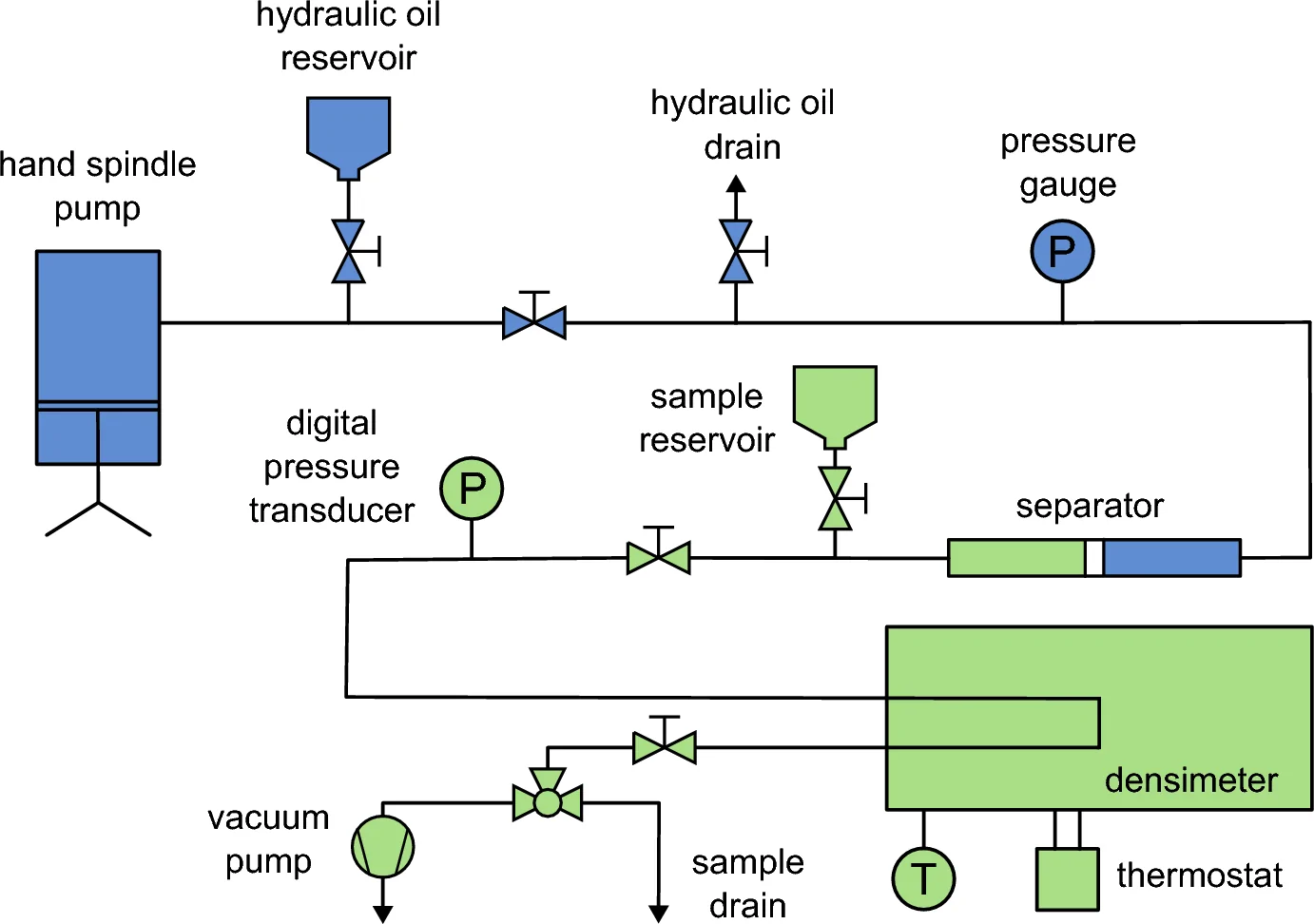
Schematic illustration of the experimental setup: the hydraulic section is highlighted in blue and the sample section is highlighted in green color

The density was measured using the DMA HPM vibrating-tube densimeter by Anton Paar together with the Anton Paar mPDS 5 evaluation unit to further process the digital data. A schematic representation of the experimental setup is shown in Fig. 1. The setup can be subdivided in two parts. First, the hydraulic section (blue color in Fig. 1), filled with mineral oil, which includes a hand spindle pump to apply pressure (monitored by an analogue pressure gauge) to the system. Second, the sample section (green color in Fig. 1), filled with the sample liquid, including the densimeter cell. A separator piston is used to transfer the pressure from the hydraulic section to the sample section. The densimeter’s temperature is controlled using a thermostat (Julabo F-32) and monitored by a built-in thermometer (Pt1000 resistance thermometer from ABB AG) with a standard uncertainty u(T)=0.1 K (specified by the manufacturer). To monitor the pressure in the sample section a digital pressure transducer (WIKA S-20) with a standard uncertainty u(p)=0.05 MPa (specified by the manufacturer) was used.

All components are specified to withstand a pressure of at least 140 MPa. To protect the components from overpressure, a safety valve in the sample part and a rupture disk in the hydraulic part (not shown in Fig. 1) were installed. All samples were degassed in the sample container using a vacuum pump before being filled into the system. During the measurement, the valves to the vacuum pump/sample drain, hydraulic oil drain, sample reservoir and hydraulic oil reservoir were closed. For every measurement, the reported value corresponds to the average of data collected over a two-minute interval.

#### Evaluation and Calibration

Different methods have been presented in the literature to calculate the density from the oscillation period of the measurement cell [45, 46]. In this work we used the calibration model by Anton Paar

1 $$\begin{aligned} \begin{aligned} \rho /\textrm{kg}\cdot \textrm{m}^{-3}&= P_{\textrm{A}} + P_{\textrm{B}} \cdot (T/\textrm{K}) + P_{\textrm{C}} \cdot (p/\textrm{MPa}) + P_{\textrm{D}} \cdot (T/\textrm{K})^2 + P_{\textrm{E}} \cdot (p/\textrm{MPa})^2 \\&\quad \ + \left( P_{\textrm{F}}+P_{\textrm{G}} \cdot (T/\textrm{K}) +P_{\textrm{H}} \cdot (p/\textrm{MPa}) +P_{\textrm{I}} \cdot (T/\textrm{K})^2 +P_{\textrm{J}} \cdot (p/\textrm{MPa})^2\right) \cdot \tau ^2\\&\quad \ + P_{\textrm{K}} \cdot \tau ^4, \end{aligned} \end{aligned}$$

where ρ is the density of the studied substance, τ the measured period, *p* the pressure, and *T* the temperature.

The device constants $P_\textrm{A} \dots P_\textrm{K}$ were determined using two calibration fluids of known density. The densities of the two calibration fluids have to be chosen so that they cover the whole expected density range of the target substances. *n*-Hexane was chosen as the low-density calibration fluid and *n*-octane as the high-density calibration fluid.

The density of the reference fluid *n*-hexane was calculated using the multiparameter EOS by *Thol et al.* [47] taken from *RefProp* [48]. The uncertainty of the homogeneous liquid densities is reported to be $u_\textrm{r}(\rho ) =$ 0.03% at atmospheric pressure, $u_\textrm{r}(\rho ) =$ 0.1% at p≤60 MPa and $263~\textrm{K} \le T \le 473~\textrm{K}$, and $u_\textrm{r}(\rho ) =$ 0.6% at higher pressures. For the density of the reference fluid *n*-octane, the multiparameter EOS from *Beckmüller et al.* [49] was used. The uncertainty of the homogeneous liquid densities is reported to be $u_\textrm{r}(\rho ) = 0.03\%$ at atmospheric pressure, and $u_\textrm{r}(\rho ) = 0.1\%$ at p≤200 MPa and $270~\textrm{K} \le T \le 440~\textrm{K}$.

The device constants were calculated from the measured data (τ,T,p) of the calibration fluids and the corresponding EOS densities, using a least-squares fit.

#### Experimental Uncertainty

The overall expanded uncertainty $U_\rho$ for the density measurement with a coverage factor k=2 (95% confidence) was calculated from the individual uncertainties as:

2 $$\begin{aligned} U(\rho ) = k \sqrt{\left( \left( \frac{\partial \rho }{\partial T}\right) _{p,\tau } u(T) \right) ^2 + \left( \left( \frac{\partial \rho }{\partial p}\right) _{T,\tau } u(p) \right) ^2 + \left( \left( \frac{\partial \rho }{\partial \tau }\right) _{T,p} u(\tau ) \right) ^2 + (u_\textrm{cal,r}~\rho )^2 }, \end{aligned}$$

with the uncertainty of the temperature *u*(*T*), the uncertainty of the pressure *u*(*p*), the uncertainty of the oscillation period u(τ), and the uncertainty of the calibration $u_\textrm{cal,r}(\rho )$. The calibration error was estimated from the uncertainty of the two EOS [47, 49], for *n*-hexane $u_\mathrm {EOS-hexane,r}$ and *n*-octane $u_\mathrm {EOS-octane,r}$, and the error $u_\textrm{fit,r}$ from the calibration fit (details are given in Supporting Information).

#### Experimental Program

For each substance, six isotherms *T*/K $\in \{293.15, 313.15,$ 333.15,353.15,373.15,393.15} were studied at 13 pressures *p*/MPa $\in \{0.1, 10, 20, 30, 40, 50,$ 60,70,80,90,100,110,120}. Only liquid states were studied. Hence, combinations that led to gas states were excluded (1-hexene for T≥353.15 K at p=0.1 MPa, 2,4-dimethylhexane for T=393.15 K at p=0.1 MPa, 2,2,4-trimethylpentane and *n*-heptane for T≥373.15 K at p=0.1 MPa).

## Modeling

### Empirical Correlation ρ(T,p)

The density data for each investigated substance were fitted to the *Tammann–Tait* equation [50], a widely used empirical approach for describing liquid densities, facilitating both the evaluation of the results and their comparison with existing literature data. The *Tammann–Tait* equation

3 $$\begin{aligned} \begin{aligned} \rho / \mathrm{kg\cdot m^{-3}} = \dfrac{\rho _0(T) / \mathrm{kg\cdot m^{-3}}}{1-e \cdot \textrm{ln}\left( \dfrac{\textrm{B}(T)+p / \textrm{MPa}}{\textrm{B}(T)+p_\textrm{ref} / \textrm{MPa}}\right) }, \end{aligned} \end{aligned}$$

accounts for the temperature dependence through two polynomial terms: the reference density,

4 $$\begin{aligned} \begin{aligned} \rho _0/\mathrm{kg\cdot m^{-3}}= c_0 + c_1 \cdot (T/\textrm{K}) + c_2 \cdot (T/\textrm{K})^2 + c_3 \cdot (T/\textrm{K})^3, \end{aligned} \end{aligned}$$

and the *B*-term,

5 $$\begin{aligned} \begin{aligned} B = d_0+ d_1 \cdot (T/\textrm{K}) + d_2 \cdot (T/\textrm{K})^2. \end{aligned} \end{aligned}$$

Together, these expressions introduce eight adjustable parameters ($c_{0-3}$, $d_{0-2}$ and *e*), with the reference pressure fixed at $p_\textrm{ref}$ = 0.1 MPa [51]. The parameters were determined individually for each substance by fitting to the experimental data using least squares. Due to the empirical nature of the correlation, the resulting parameterizations are not suitable for extrapolation beyond the measured range. The fitted parameter values are provided in the Supporting Information.

### Molecular-Based Equation of State

Many modern molecular-based EOSs are based on the statistical associating fluid theory, SAFT [52] e.g., PC-SAFT [53, 54], soft-SAFT [55], group-contribution SAFT [30], SAFT-VR Mie [41]. SAFT is based on *Wertheim’s* thermodynamic perturbation theory [56–59] and does not constitute a specific EOS, but rather a framework that enables the integration of specific molecular interaction and architecture features into a given theoretical model [27]. Previous studies have shown that the SAFT-VR Mie EOS developed by *Lafitte et al.* [41] provides reliable predictions, particularly at high pressures and high temperatures [33, 34, 42, 43]. For this reason, the SAFT-VR Mie EOS was employed in this work to model the liquid densities of the studied fluids.

In contrast to other SAFT-type EOS, the SAFT-VR Mie employs variable-range (VR) intermolecular interactions together with the Mie potential to describe both repulsive and attractive forces between molecules [41]. The Mie potential is defined as [60, 61]

6 $$\begin{aligned} U_\textrm{Mie} = \varepsilon \left( \frac{\lambda _\textrm{r}}{\lambda _\textrm{r} - \lambda _\textrm{a}} \right) \left( \frac{\lambda _\textrm{r}}{\lambda _\textrm{a}} \right) ^{\frac{\lambda _\textrm{a}}{\lambda _\textrm{r} - \lambda _\textrm{a}}} \left[ \left( \frac{\sigma }{r} \right) ^{\lambda _\textrm{r}} - \left( \frac{\sigma }{r} \right) ^{\lambda _\textrm{a}} \right] , \end{aligned}$$

where *r* denotes the distance between the sphere segments, σ is the segment diameter, and ε represents the depth of the potential well. The exponents $\lambda _\textrm{a}$ and $\lambda _\textrm{r}$ correspond to the attractive and repulsive exponents, respectively. The SAFT-VR Mie EOS models the *Helmholtz* energy

7 $$\begin{aligned} a = a_\textrm{id} + a_\textrm{mono} + a_\textrm{chain} + a_\textrm{assoc}, \end{aligned}$$

as the sum of contributions for the ideal gas $a_\textrm{id}$, a monomer contribution for Mie fluids $a_\textrm{mono}$, a chain contribution $a_\textrm{chain}$, and an association contribution for the bonding between Mie segments $a_\textrm{assoc}$. The terms are described in detail in the work by *Lafitte et al.* [41].

The parameters used for the SAFT-VR Mie EOS were adopted from *Dänekas* [62], who optimized them by fitting to experimental data for vapor pressures, saturated densities, enthalpies of vaporization, heat capacities, and speeds of sound; the compressed-liquid density data reported in this work were not included in the parameter fit. The parameter sets are given in Table 2.

**Table 2:** SAFT-VR Mie component-specific model parameters from *Dänekas* [62]: the columns give the segment number *m*, segment diameter σ, segment dispersion energy ε, and repulsive exponent $\lambda _{\mathrm r}$

Substance	*m*	σ (Å)	$\varepsilon /k_{\mathrm B}{\mathrm K}$	$\lambda _{\mathrm r}$
1-Hexene	1.9918	4.4468	359.7090	17.0677
2,4-Dimethylhexane	2.2929	4.6884	388.3900	18.6570
2,2,4-Trimethylpentane	2.3566	5.1446	445.2066	21.4209
*n*-Heptane	2.4991	4.4507	362.4290	17.2519

The attractive exponent was $\lambda _{a} = 6$ in all cases

### Evaluation of the Models

To quantify the deviations between the experimental data and a given model—either the empirical correlation or the EOS—the average absolute deviation (AAD)8is employed in this work. The experimental data are denoted by $\rho _{\textrm{exp}}^\textrm{src}$, where the source index (src) indicates whether the data originate from this work (th) or from the literature (lit). The corresponding model values are $\rho _{\textrm{mod},i}$, and refer to results either from the empirical correlation (corr) or from the equation of state (EOS).

## Results and Discussion

### Experimental Results

The experimental density data are given in Table 3.

**Table 3:** Measured densities of 1-hexene, 2,4-dimethylhexane, 2,2,4-trimethylpentane, and *n*-heptane

*p* (MPa)	$\rho \,(\textrm{kg}\cdot \mathrm {m^{-3}})$	*p* (MPa)	$\rho \, (\textrm{kg}\cdot \mathrm {m^{-3}})$	*p* (MPa)	$\rho \, (\textrm{kg}\cdot \mathrm {m^{-3}})$
1-Hexene					
T=293.15 K		T=313.15 K		T=333.15 K	
0.09	673.06	0.09	654.34	0.11	634.35
10.10	683.23	10.11	666.13	10.10	648.14
20.10	692.20	20.10	676.26	20.10	659.76
30.10	700.27	30.10	685.16	30.10	669.70
40.10	707.57	40.10	693.27	40.10	678.59
50.10	714.33	50.09	700.63	50.10	686.64
60.10	720.62	60.10	707.41	60.10	694.10
70.10	726.46	70.10	713.74	70.10	700.89
80.10	731.93	80.10	719.61	80.11	707.18
90.09	737.14	90.11	725.16	90.10	713.14
100.10	742.09	100.11	730.38	100.10	718.72
110.11	746.78	110.11	735.39	110.11	723.97
120.09	751.75	120.11	740.12	120.09	728.97

T=353.15 K		T=373.15 K		T=393.15 K	
10.11	630.14	10.10	611.60	10.11	592.07
20.11	643.33	20.10	626.89	20.11	609.83
30.10	654.57	30.11	639.48	30.11	624.05
40.10	664.28	40.10	650.35	40.10	635.95
50.10	673.05	50.10	659.91	50.10	646.49
60.10	681.01	60.10	668.48	60.11	655.88
70.10	688.33	70.10	676.32	70.10	664.25
80.11	695.08	80.10	683.59	80.11	671.97
90.10	701.52	90.09	690.38	90.11	679.07
100.10	707.40	100.11	696.63	100.11	685.75
110.11	712.98	110.11	702.57	110.11	692.07
120.09	718.28	120.09	708.06	120.08	697.83

2,4-Dimethylhexane					
T=293.15 K		T=313.15 K		T=333.15 K	
0.11	699.79	0.10	683.79	0.09	666.79
10.10	708.55	10.10	693.88	10.11	678.37
20.10	716.53	20.09	702.72	20.11	688.31
30.10	723.65	30.10	710.58	30.10	697.01
40.10	730.38	40.10	717.77	40.11	704.90
50.10	736.38	50.10	724.33	50.10	712.03
60.10	742.02	60.10	730.42	60.10	718.65
70.10	747.40	70.10	736.16	70.10	724.74
80.10	752.44	80.09	741.45	80.09	730.44
90.09	757.12	90.10	746.54	90.09	735.79
100.10	761.67	100.11	751.33	100.11	740.79
110.09	765.87	110.11	755.85	110.10	745.60
120.11	770.06	120.09	760.13	120.09	750.16
T=353.15 K		T=373.15 K		T=393.15 K	
0.07	648.83	0.09	630.55		
10.11	662.19	10.09	645.86	10.11	629.43
20.10	673.37	20.10	658.57	20.11	643.66
30.08	683.10	30.09	669.30	30.08	655.63
40.10	691.66	40.10	678.72	40.10	665.91
50.10	699.40	50.10	687.12	50.10	675.15
60.10	706.49	60.10	694.65	60.12	683.61
70.10	713.02	70.11	701.74	70.10	690.91
80.09	719.18	80.11	708.20	80.10	698.04
90.09	724.87	90.09	714.25	90.11	704.34
100.10	730.18	100.10	719.95	100.12	710.30
110.11	735.26	110.10	725.35	110.09	715.90
120.11	740.05	120.09	730.36	120.10	721.40

2,2,4-Trimethylpentane					
T=293.15 K		T=313.15 K		T=333.15 K	
0.09	691.74	0.09	675.30	0.11	658.39
10.10	701.36	10.11	686.21	10.11	670.83
20.10	709.72	20.10	695.56	20.11	681.30
30.10	717.26	30.10	703.89	30.10	690.54
40.10	724.12	40.10	711.36	40.10	698.66
50.10	730.42	50.10	718.18	50.10	706.08
60.09	736.33	60.10	724.51	60.10	712.88
70.10	741.94	70.10	730.39	70.10	719.15
80.10	747.09	80.09	735.93	80.11	725.00
90.09	751.91	90.09	741.08	90.09	730.52
100.10	756.51	100.11	746.01	100.10	735.66
110.10	760.92	110.10	750.65	110.11	740.54
120.10	765.14	120.10	755.01	120.10	745.18
T=353.15 K		T=373.15 K		T=393.15 K	
0.09	640.66				
10.11	655.21	10.11	639.53	10.11	623.23
20.10	667.03	20.09	652.82	20.10	638.36
30.10	677.28	30.10	664.10	30.10	650.85
40.10	686.23	40.10	673.99	40.10	661.54
50.10	694.26	50.10	682.64	50.10	670.98
60.10	701.62	60.11	690.50	60.10	679.43

T=353.15 K		T=373.15 K		T=393.15 K	
70.10	708.32	70.10	697.57	70.10	687.09
80.11	714.57	80.10	704.33	80.10	694.17
90.09	720.40	90.10	710.53	90.10	700.81
100.09	725.87	100.09	716.35	100.09	706.88
110.09	731.00	110.09	721.80	110.10	712.65
120.08	735.81	120.08	727.01	120.11	718.00

*n*-Heptane					
T=293.15 K		T=313.15 K		T=333.15 K	
0.10	684.21	0.10	666.94	0.09	649.43
10.10	693.14	10.11	677.25	10.10	661.25
20.10	701.15	20.10	686.21	20.09	671.36
30.09	708.36	30.11	694.17	30.10	680.18
40.10	715.00	40.10	701.40	40.10	688.16
50.10	721.39	50.10	708.06	50.11	695.43
60.09	727.17	60.10	714.27	60.10	702.08
70.10	732.44	70.10	719.95	70.10	708.30
80.11	737.43	80.11	725.36	80.10	714.03
90.09	742.29	90.10	730.43	90.09	719.48
100.10	746.73	100.10	735.30	100.09	724.62
110.11	751.11	110.10	739.93	110.11	729.45
120.11	755.25	120.08	744.26	120.10	734.07
T=353.15 K		T=373.15 K		T=393.15 K	
0.10	631.33				
10.10	644.99	10.11	628.68	10.11	611.45
20.11	656.41	20.10	641.58	20.11	626.17
30.10	666.27	30.10	652.36	30.10	638.40
40.10	675.06	40.09	661.90	40.10	648.90
50.10	682.88	50.10	670.52	50.10	658.20
60.10	690.09	60.10	678.29	60.12	666.51
70.10	696.73	70.09	685.47	70.10	674.28
80.09	702.89	80.11	692.01	80.10	681.30
90.10	708.70	90.11	698.26	90.10	687.83
100.10	714.25	100.10	704.04	100.10	693.97
110.10	719.40	110.10	709.47	110.11	699.64
120.09	724.28	120.09	714.55	120.10	705.14

The standard uncertainties of the temperature *T* and pressure *p* are u(T)=0.1 K and u(p)=0.05 MPa, respectively. The combined expanded uncertainty (k=2) for the density is $U(\rho ) = 1.52~{\textrm{kg}\cdot \mathrm {m^{-3}}}$ for 1-hexene, $U(\rho ) = 1.56~{\textrm{kg}\cdot \mathrm {m^{-3}}}$ for 2,4-dimethylhexane, $U(\rho ) = 1.55~{\textrm{kg}\cdot \mathrm {m^{-3}}}$ for 2,2,4-trimethylpentane, and $U(\rho ) = 1.53~{\textrm{kg}\cdot \mathrm {m^{-3}}}$ for *n*-heptane, corresponding roughly to 0.2%

**Fig. 2:**
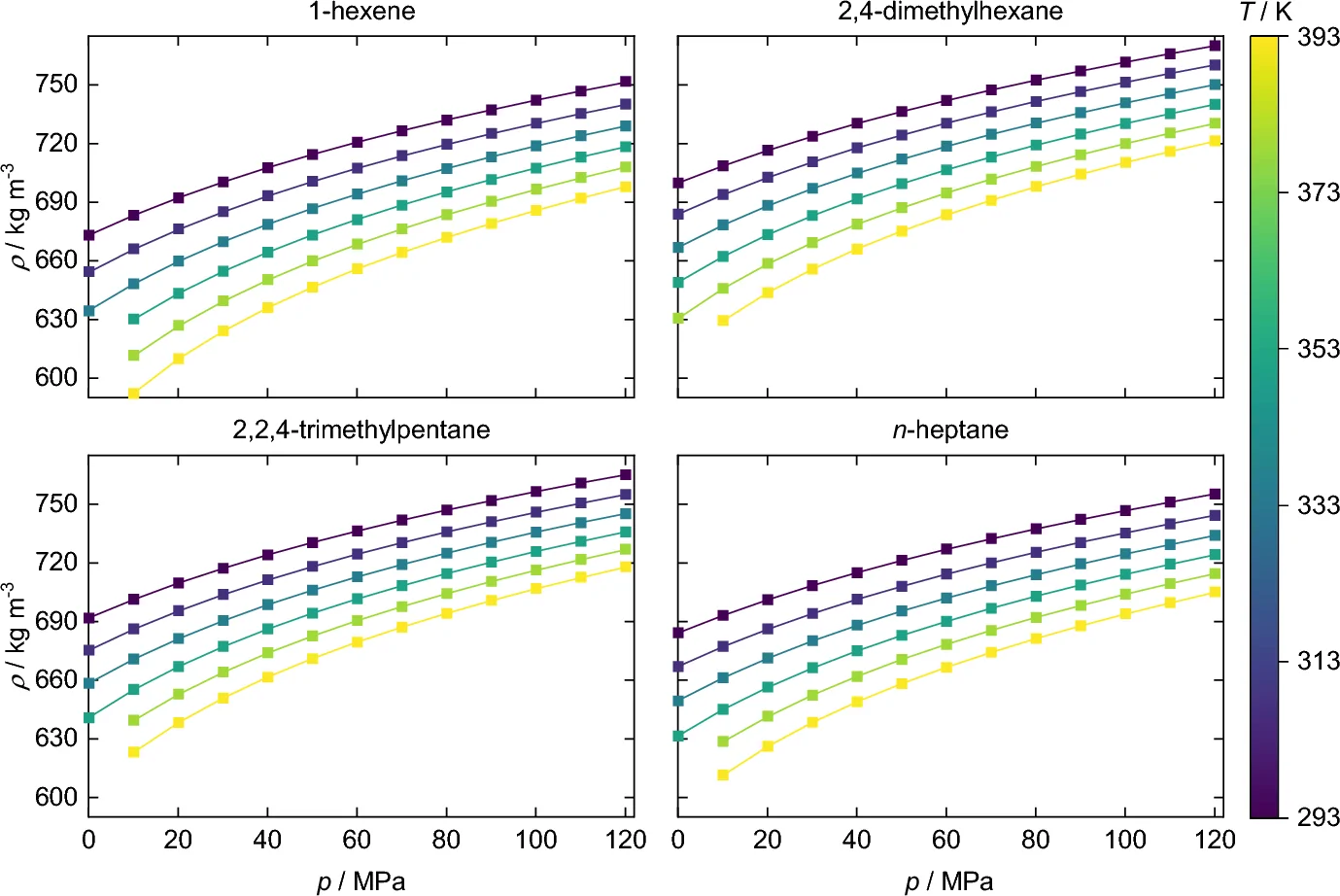
Density ρ of the studied hydrocarbons as a function of pressure *p*. Symbols: experimental results from this work; lines: *Tammann–Tait* correlation (Eq. 3). The color indicates the temperature

Figure 2 shows the experimental results from this work together with the results from the empirical correlation as a function of the pressure. The parameters for the component specific, empirical correlations are given in the Supporting Information. The empirical correlations describe the experimental data well, as discussed below in more detail.

As expected, for all studied substances, the density increases with increasing pressure and decreasing temperature. 1-Hexene has the lowest density across the examined temperature and pressure range, while 2,4-dimethylhexane has the largest density. However, the differences in the density between the studied substances are comparatively small and do not exceed 3%, which is considerably below the differences resulting from the variation of temperature or pressure in the ranges studied here.

**Fig. 3:**
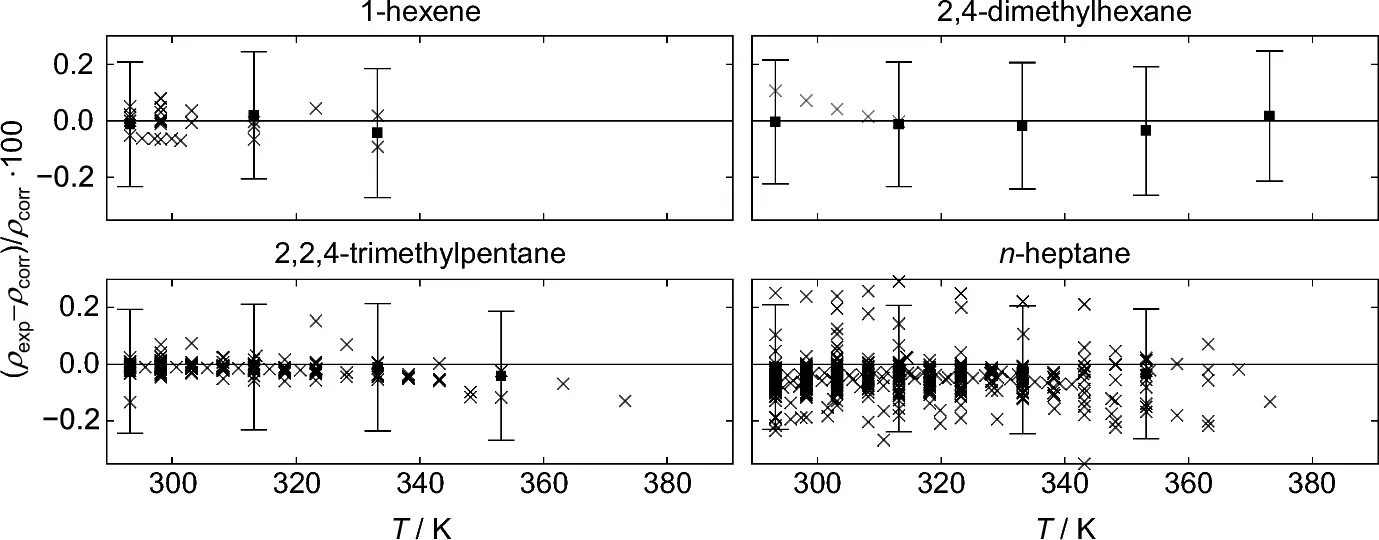
Relative deviations between experimental density data (symbols) and the empirical correlations (cf. Eq. 3) (baseline) at ambient pressure as a function of the temperature *T*. Solid squares indicate results from this work [with error bars U(ρ) (k=2)], and black crosses indicate literature data [20, 63–312]

An overview of the available literature data for all studied hydrocarbons is given in the Supporting Information. To compare the results from the present work with those data a representation is used that takes the individual *Tammann–Tait* correlations as reference and presents the relative deviations of the literature data as well as the data from the present work relative to that reference. Figure 3 presents the results for ambient pressure where literature data are most plentiful. For the data from the present work, error bars are also shown; they represent a conservative estimate of the uncertainty and are about ± 0.2% for all data. The uncertainties of the literature data, which are only partly available, are not shown in Fig. 3 for clarity of presentation. Most of the literature data agree with the present data within 0.2%, typical deviations are below 0.1%. The deviation of the present results from the correlations, which were fitted to these data, is below 0.05% for all substances. For *n*-heptane, plenty of literature data are available at ambient pressure. They scatter around the correlation in a band of ± 0.3%. Figure 3 also shows that the deviations do not depend systematically on the temperature.

**Fig. 4:**
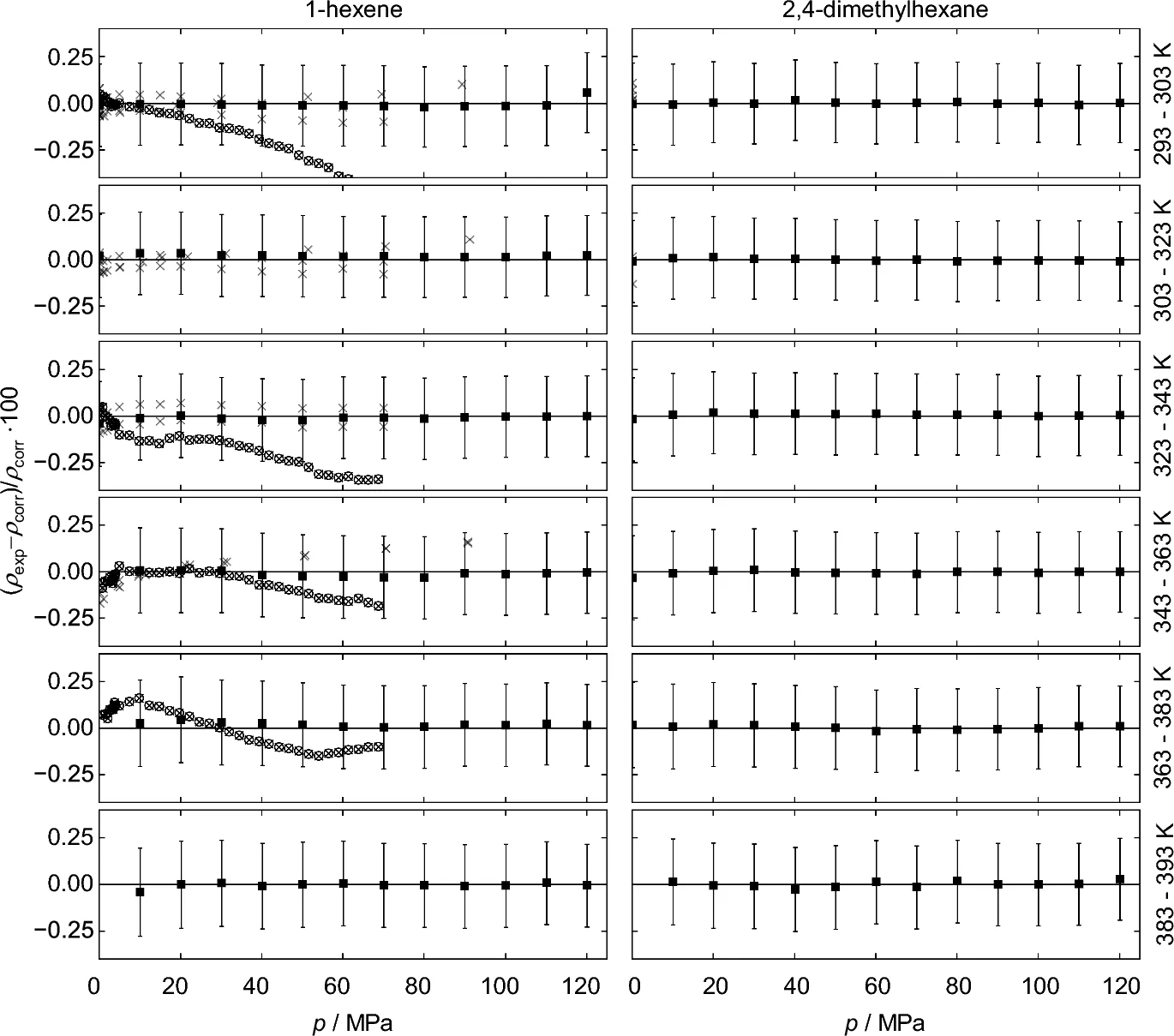
Relative deviations between experimental density data (symbols) and the empirical correlations (cf. Eq. 3) (baseline) for 1-hexene and 2,4-dimethylhexane as a function of the pressure *p*. Data for temperatures in different intervals are given in separate panels and compared to the corresponding correlation at that temperature. Solid squares indicate results from this work [with error bars U(ρ) (k=2)], and black crosses indicate literature data [4, 306, 307, 313–322]; the dataset by *Kerimov and Apaev* [313] is additionally highlighted by open circles

**Fig. 5:**
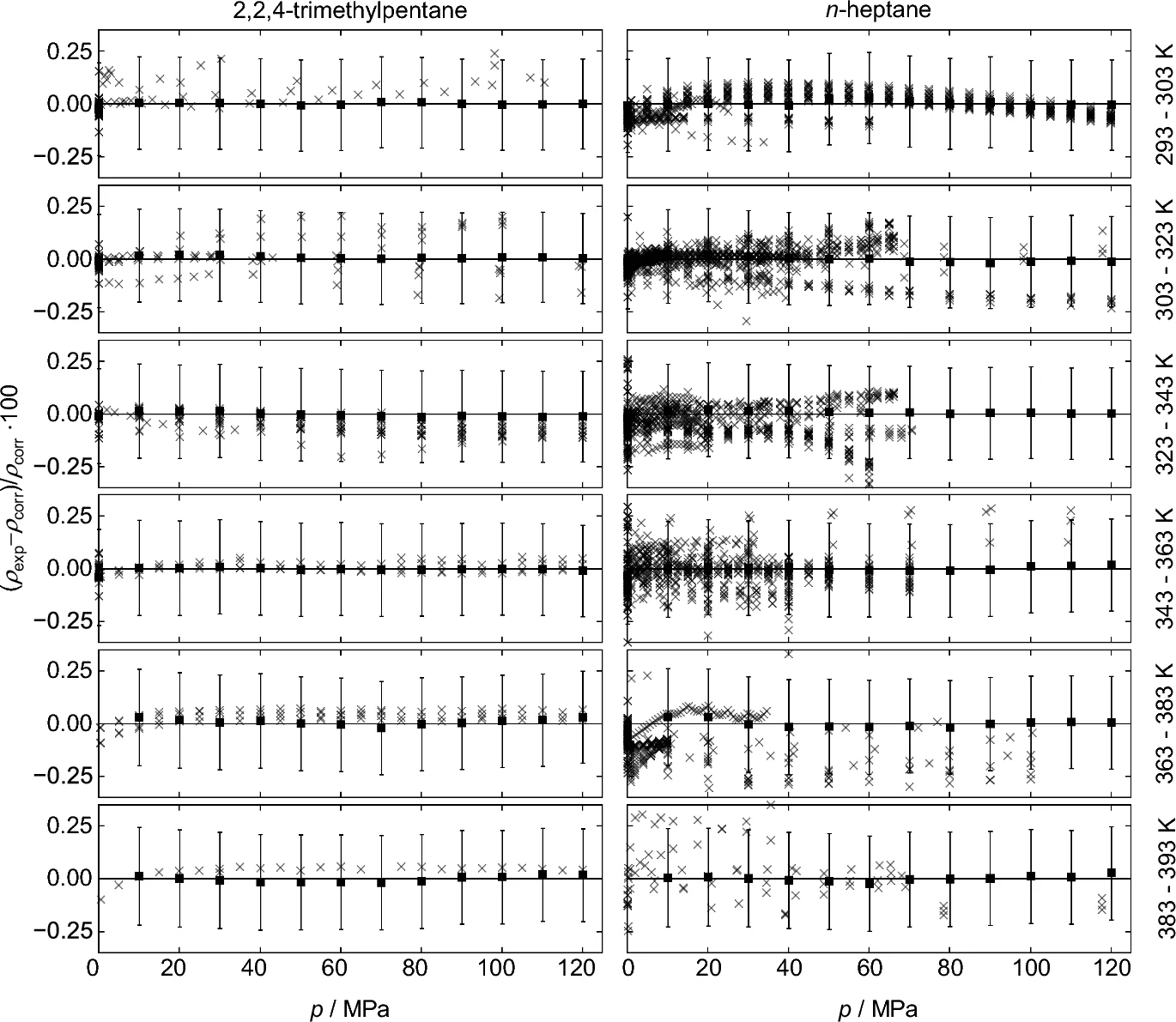
Relative deviations between experimental density data (symbols) and the empirical correlations (cf. Eq. 3) (baseline) for 2,2,4-trimethylpentane and *n*-heptane as a function of the pressure *p*. Data for temperatures in different intervals are given in separate panels and compared to the corresponding correlation at that temperature. Solid squares indicate results from this work [with error bars U(ρ) (k=2)], and black crosses indicate literature data [20, 63–312]

Figures 4 and 5 show the relative deviations of the literature data and the new experimental data from the correlations (Eq. 3) as a function of pressure along isotherms; Fig. 4 covers 1-hexene and 2,4-dimethylhexane, and Fig. 5 covers 2,2,4-trimethylpentane and *n*-heptane. For all substances, the new data agree excellently with the respective correlations.

For 1-hexene (Fig. 4), the data from *Kerimov and Apaev* [313], show systematic deviations from the present results, increasing with pressure. These deviations mostly lie outside the uncertainty of only 0.05% reported by *Kerimov and Apaev* [313], and, at the highest pressures, also outside the conservative uncertainty estimate of 0.2% for our results. Since the other literature data agree well with our results, we question the uncertainty stated by *Kerimov and Apaev* [313]. For 2,4-dimethylhexane (Fig. 4), no literature data at elevated pressures are available for comparison with the present results. For 2,2,4-trimethylpentane (Fig. 5), the deviations of the available literature data from the present results lie within our reported uncertainty. The deviations occur in both directions, supporting the overall quality of our results. A large amount of liquid density data at elevated pressures has been published for *n*-heptane (Fig. 5), producing scatter that exceeds the reported uncertainties. Nevertheless, most of the literature data lie within the error bars of the present results. Again, deviations occur in both directions, supporting our results.

Table 4 summarizes the deviations from the correlations in terms of the AAD for each substance, both for the present results and for the literature data.

**Table 4:** Average absolute deviations (AAD, Eq. 8) of the literature data (lit) and the experimental data from this work (th) from the *Tammann–Tait* correlations (Eq. 3)

Substance	$\textrm{AAD}_{\rho ,\textrm{corr}}^\textrm{lit}\, (\%)$	$\textrm{AAD}_{\rho ,\textrm{corr}}^\textrm{th}\,(\%)$
1-Hexene	0.075	0.015
2,4-Dimethylhexane	0.056	0.009
2,2,4-Trimethylpentane	0.040	0.010
*n*-Heptane	0.065	0.010

The columns indicate (left to right) the substance name, the average deviations $\textrm{AAD}_{\rho ,\textrm{corr}}^\textrm{lit}$ from the literature data, and the average deviations $\textrm{AAD}_{\rho ,\textrm{corr}}^\textrm{th}$ from the measurement data from this work (cf. Eq. 8)

### EOS Modeling Results

In this section, the SAFT-VR Mie EOS [41] is evaluated as a predictor of the measured liquid densities. The component-specific parameters from *Dänekas* [62] were obtained by fitting to previously available vapor pressures, densities, and caloric data. The present comparison therefore probes the EOS performance in a state region outside its parameterization domain.

**Fig. 6:**
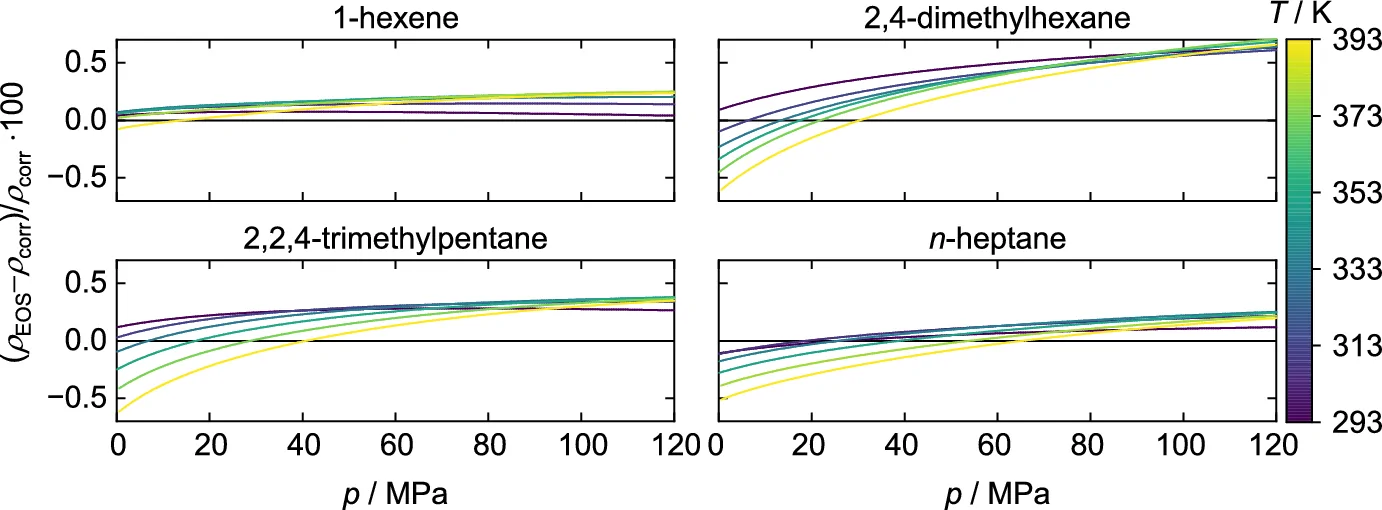
Deviations between the density results from the SAFT-VR Mie EOS [41] and the *Tammann–Tait* correlations (cf. Eq. 3) (baseline) for all four studied substances and the six investigated isotherms as a function of pressure *p*. The color indicates the temperature

Figure 6 shows the relative deviations between the densities from the SAFT-VR Mie EOS [41] and the *Tammann–Tait* correlations (cf. Eq. 3), which serve as the reference here for all four substances. Across all substances, the EOS slightly overestimates the density at high pressures; the deviations decrease for lower pressures turning negative for most of the studied isotherms. The largest deviations occur for the two branched alkanes, 2,4-dimethylhexane and 2,2,4-trimethylpentane, reaching up to about 0.7%. This can be attributed to the molecular model underlying SAFT-VR Mie, in which a molecule is represented as a linear chain of tangentially bonded spherical segments. Molecular architecture, such as branching, is not resolved explicitly by the chain topology and enters the model only implicitly through the fitted parameters [323, 324]. Consequently, deviations from experimental data are expected to be larger for branched alkanes than for linear ones.

The deviations between the EOS predictions and both the present data (th) and the literature data (lit) are summarized in Table 5. For *n*-heptane and 1-hexene, the AAD values lie between 0.13% and 0.17% and agree closely between the two data sources. For the branched alkanes, the AAD values are higher and the two sources diverge. For 2,4-dimethylhexane, the AAD for the new data is 0.38% as compared to that for the literature data of 0.06%. This reflects the fact that literature data are only available at low pressures, whereas the present data extend the comparison to 120 MPa. In contrast, for 2,2,4-trimethylpentane, the EOS describes the present data (0.24%) more accurately than the literature data (0.38%). Since neither dataset entered the parameterization, the closer agreement with the present data indicates that the multi-property fit, anchored in vapor–liquid equilibrium and caloric properties, projects into the compressed-liquid region in a way that is consistent with the new measurements—as expected from a consistent molecular-based EOS. The increased deviation for the literature data is probably related to their large scattering. Overall, the AAD values lie in the range of 0.13% to 0.38%, on the order of the expanded experimental uncertainty and in line with what a multi-property parameterization can be expected to deliver outside its fitting domain.

**Table 5:** Average absolute deviations (AAD, Eq. 8) of the literature data (lit) and the experimental data from this work (th) from the SAFT-VR Mie EOS

Substance	$\textrm{AAD}_{\rho ,\textrm{EOS}}^\textrm{th} \, (\%)$	$\textrm{AAD}_{\rho ,\textrm{EOS}}^\textrm{lit} \, (\%)$
1-Hexene	0.17	0.17
2,4-Dimethylhexane	0.38	0.06
2,2,4-Trimethylpentane	0.24	0.38
*n*-Heptane	0.13	0.15

The columns indicate (left to right) the substance name, the average deviations $\textrm{AAD}_{\rho ,\textrm{EOS}}^\textrm{th}$ and $\textrm{AAD}_{\rho ,\textrm{EOS}}^\textrm{lit}$ (cf. Eq. 8)

## Conclusions

In this work, new density data for the four hydrocarbons 1-hexene, 2,4-dimethylhexane, 2,2,4-trimethylpentane, and *n*-heptane are reported for six isotherms in the range 293.15 K to 393.15 K and at pressures up to 120 MPa. These substances are technically relevant as fuel components and primary reference fuels for gasoline knock ratings, as the co-monomer in polyethylene production (1-hexene), and as model compounds for the development of equations of state for linear and branched alkanes.

The expanded relative uncertainty of the measurements is approximately 0.2% (*k* = 2). This is a conservative estimate: the new data are described by the *Tammann–Tait* correlations established in this work with an average absolute deviation of only 0.01%, and reliable literature data also lie almost entirely within these uncertainty bounds. Both observations indicate that the true accuracy of the new measurements is substantially better than the stated value.

The new data also clarify the literature situation for the four hydrocarbons studied here. For 2,4-dimethylhexane, only a single source reporting ambient-pressure values was previously available; the present work therefore provides the first high-pressure dataset for this substance. For 1-hexene, comparison with the new data indicates that the previously reported high-pressure dataset of *Kerimov and Apaev* [313] is questionable. For 2,2,4-trimethylpentane and *n*-heptane, the new measurements lie centrally within the scatter of an extensive set of literature data and thereby corroborate the consistency of those data.

The SAFT-VR Mie equation of state was deployed for modeling the new data using a parameter set that was adjusted to a broad range of thermodynamic properties without specific emphasis on compressed-liquid densities. The overall deviations are on the order of the expanded experimental uncertainty, with the largest deviations observed for the two branched alkanes. This is consistent with the SAFT-VR Mie molecular model, in which a molecule is represented as a linear chain of tangentially bonded spherical segments; molecular architecture such as branching is therefore not resolved explicitly, but only enters implicitly through the fitted parameters. The new data and correlations thus provide both a reliable reference for liquid hydrocarbon densities over a wide range of temperatures and pressures and a benchmark for the targeted improvement of molecular-based equations of state for branched alkanes.

## Supplementary Information

Below is the link to the electronic supplementary material.

Supplementary file 1 (pdf 589 KB)

## Data Availability

The data from this study are provided in the present manuscript.
