# Supplementary material for: High-Pressure Density Measurements and Modeling of Hydrocarbons: 1-Hexene, 2,4-Dimethylhexane, 2,2,4-Trimethylpentane, and n-Heptane
Source: Int J Thermophys. 2026 Sep 6;47(9):157. doi: 10.1007/s10765-026-03823-6 (PMC13547189; doi:10.1007/s10765-026-03823-6)
Supplement: Supplementary file 1 [file 10765_2026_3823_MOESM1_ESM.pdf]

Supporting Information: High-Pressure Density Measurements and Modeling of Hydrocarbons: 1-hexene, 2,4-dimethylhexane, 2,2,4-trimethylpentane, and *n*-heptane

Dennis Alt,† Bennet Hopp,† Jens Staubach,†,‡ Hans Hasse,¶ and Simon Stephan*,†,‡

†*Molecular Thermodynamics Group (MTG), RPTU Kaiserslautern, Kaiserslautern 67663, Germany*

‡*Laboratory of Heat and Mass Transfer (WSÜ), OVGU Magdeburg, Magdeburg 39106, Germany*

¶*Laboratory of Engineering Thermodynamics (LTD), RPTU Kaiserslautern, Kaiserslautern 67663, Germany*

E-mail: simon.stephan@ovgu.de

State Points

Fig. S1 displays the state points at which density data for the investigated substances were available in the Dortmund Data Bank (DDB)[S1] within the temperature and pressure range considered in this work. Only data for which the primary literature source was accessible were included in the analysis.

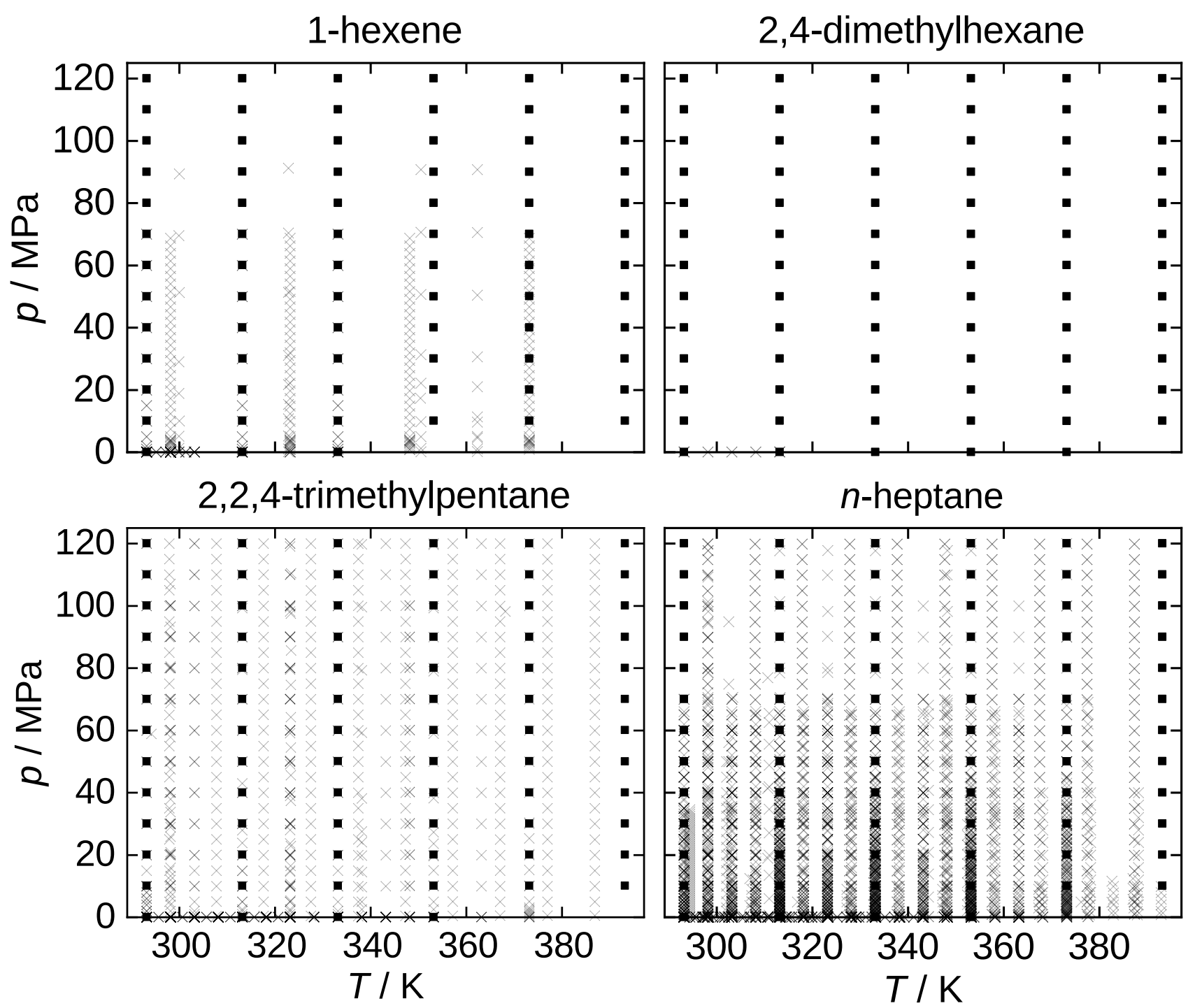

Figure S1: Pressures and temperatures for which data on the density of 1-hexene, 2,4-dimethylhexane, 2,2,4-trimethylpentane, and *n*-heptane were available for temperatures up to 393 K: literature data[S1] (crosses) and experiments from this work (squares).

Multi-parameter EOS of Calibration Fluids

Fig. S2 shows the deviation of the EOS used for calibration[S2,S3] from the literature data taken from DDB[S1] of *n*-hexane and *n*-octane.

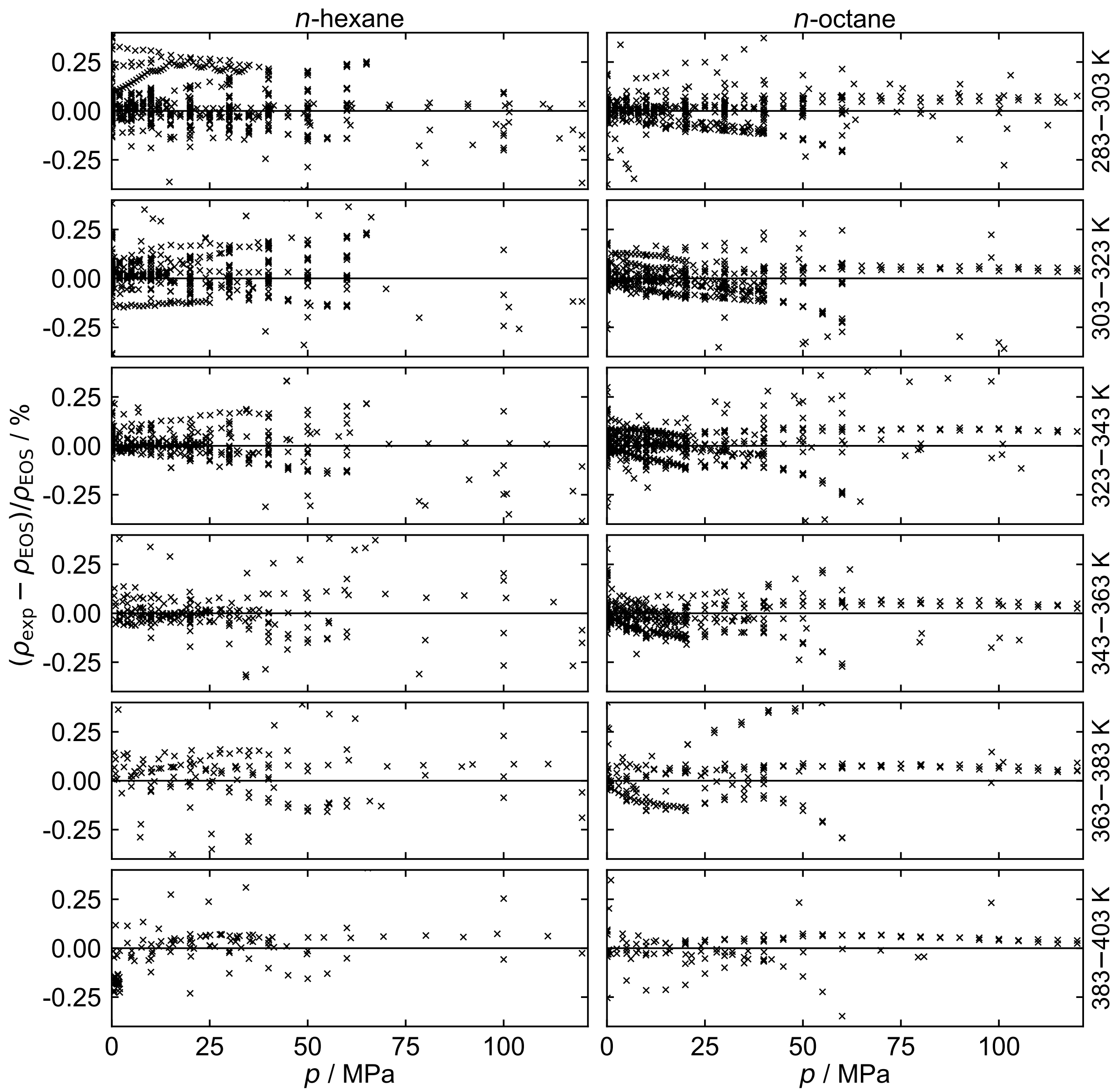

Figure S2: Relative deviations of experimental data and results from the multiparameter EOS as a function of the pressure p for the reference fluids n-hexane and n-octane. Experimental data ρ_{exp} were taken from the DDB.[S1] The EOS from *Beckmüller et al.*[S3](*n*-octane) and *Thol et al.*[S2,S4] (*n*-hexane) were used to calculate the densities ρ_{EOS}.

The average absolute deviation between the experimental data provided by DDB[S1] and the EOS are given in Table S1. In the case of n-hexane at high temperatures and low pressures, the EOS yields vapor phase densities for some state points that were still liquid according to the literature data. These values were not considered when calculating the

$AAD^{lit}_{\rho,EOS}$. For the state points required for calibration, the EOS only yields liquid densities.

Table S1: Deviations of the experimental data taken from DDB[S1] and the EOS[S2–S4] which were used for calibration. The columns indicate (left to right) the substance name, the average deviations $AAD^{lit}_{\rho,EOS}$ (cf. Eq. 8 in the main body of this work).

substance	$AAD^{lit}_{\rho,EOS}$ / %
n-hexane	0.11
n-octane	0.11

Estimation of the Calibration Uncertainty

The relative standard calibration uncertainty $u_{cal,r}(\rho)$ was estimated as described by *Schmitt et al.*:[S5]

$$u_{cal,r}(\rho) = \sqrt{u^2_{fit,r} + u^2_{EOS-hexane,r} + u^2_{EOS-octane,r}}. \tag{S1}$$

With the standard uncertainty of the calibration fit $u_{fit,r} = 0.00022$ and the standard uncertainties for the data $u_{EOS-hexane,r} = 0.00071$ and $u_{EOS-octane,r} = 0.00048$. These are smaller than the previously calculated AAD values because the data were filtered to remove the influence of outliers and to get a true estimate of the uncertainty. The uncertainty of the calibration is therefore $u_{cal,r}(\rho) = 0.00088$.

Parameters of Empirical Correlations $\rho_{corr}(T, p)$

Table S2 shows the parameters of the empirical correlation for the respective hydrocarbons. These were obtained from the measured data using a least-squares fit. The empirical correlation is only valid for the respective measured pressure and temperature range. The average absolute deviations of the empirical correlations from the data from this work $AAD^{th}_{\rho,corr}$ and from the literature data $AAD^{lit}_{\rho,corr}$ are given in Table 4 in the main body of this work.

Table S2: Parameters for the Tammann-Tait correlations $\rho_{\mathrm{corr}}(T, p)$ (cf. Eq. 3 in the main body of this work) for the hydrocarbons studied in this work.

	1-hexene	2,4-dimethylhexane	2,2,4-trimethylpentane	*n*-heptane
c_0	1027.323881	748.5024654	1001.687804	1092.673628
c_1	-1.931211592	0.636549681	-1.60849159	-2.443294247
c_2	0.003992164	-0.003413227	0.002957899	0.00534707
c_3	-5.20E-06	2.30E-06	-3.67E-06	-6.02E-06
d_0	307.5687387	299.9024489	287.4458549	312.3269664
d_1	-1.202051604	-1.091812013	-1.07834978	-1.167459954
d_2	0.00118767	0.001003396	0.001031326	0.001109096
e	0.092171356	0.088166582	0.087155364	0.090325645

References

(S1) Dortmund Data Bank. 2024; https://www.ddbst.com.

(S2) Bell, I. H.; Wronski, J.; Quoilin, S.; Lemort, V. Pure and Pseudo-pure Fluid Thermophysical Property Evaluation and the Open-Source Thermophysical Property Library CoolProp. *Ind. Eng. Chem. Res.* **2014**, *53*, 2498–2508.

(S3) Beckmüller, R.; Span, R.; Lemmon, E.; Thol, M. A Fundamental Equation of State for the Calculation of Thermodynamic Properties of n-Octane. *J. Phys. Chem. Ref. Data* **2022**, *51*, 043103.

(S4) Thol, M.; Wang, Y.; Lemmon, E. W.; Span, R. Fundamental Equations of State for Hydrocarbons. Part II. n-Hexane. **2019**,

(S5) Schmitt, S.; Hasse, H.; Stephan, S. Measurements and Equation of State Modeling of the Density of Five 1-Alcohols (C6–C10) at Pressures of up to 120 MPa. *J. Chem. Eng. Data* **2024**, *69*, 2967–2983.
